# Population Structure and the Colonization Route of One of the Oldest North American Invasive Insects: Stories from the Worn Road of the Hessian Fly, *Mayetiola destructor* (Say)

**DOI:** 10.1371/journal.pone.0059833

**Published:** 2013-03-27

**Authors:** Philip K. Morton, Brandon J. Schemerhorn

**Affiliations:** 1 Department of Entomology, Purdue University, West Lafayette, Indiana, United States of America; 2 USDA-ARS, West Lafayette, Indiana, United States of America; University of Guelph, Canada

## Abstract

An integral part to understanding the biology of an invasive species is determining its origin, particularly in pest species. As one of the oldest known invasive species, the goals of this study were to evaluate the evidence of a westward expansion of Hessian fly into North America, from a potential singular introduction event, and the population genetic structure of current populations. Levels of genetic diversity and population structure in the Hessian fly were compared across North America, Europe, North Africa, Western Asia, and New Zealand. Furthermore, Old World populations were evaluated as possible sources of introduction. We tested diversity and population structure by examining 18 microsatellite loci with coverage across all four Hessian fly chromosomes. Neither genetic diversity nor population genetic structure provided evidence of a westward movement from a single introduction in North America. Introduced populations in North America did not show identity or assignment to any Old World population, likely indicating a multiple introduction scenario with subsequent gene flow between populations. Diversity and selection were assessed on a chromosomal level, with no differences in diversity or selection between chromosomes or between native and introduced populations.

## Introduction

Invasive species, particularly insects, can have damaging consequences to natural environments and agro-ecosystems [1], [2]. Part of understanding the biology of these pest insects is determining their origin to reduce the potential for future invasions, establish a control program for an organism, or at a minimum, to develop hypotheses on their ecology and evolution. This has led to investigations in the population origin of such insects as the red imported fire ant [3], corn rootworm [4], and emerald ash borer [5]. However, all these insects are relatively recent invaders. Uncovering the history from an invasion >200 years since the initial introduction may prove to be more difficult. Understanding the path and history of an invasion of this age can still provide information to base additional hypotheses or provide information on routes of future invasions, which could add to management or prevention plans.

First reported in North America in 1779, the Hessian fly, *Mayetiola destructor* (Say), is an introduced wheat pest, where it is one of the oldest known invasive species [6]. It is hypothesized that the North American introduction originated from straw bedding brought from Europe by Hessian mercenaries who arrived in New York during the Revolutionary War, which was also the site of the first reported Hessian fly infestation [6], [7], [8], [9]. Prevailing Hessian fly history claims this introduction was singularly responsible for all current populations of Hessian fly in North America. After introduction, historic accounts claim a southerly, and then westward colonization route, across most of North America [7], [8]. Packard [10] offered an additional hypothesis of a second introduction of Hessian fly into California via Spanish settlers. Today, Hessian fly occupies most of the wheat growing regions in the United States [11], [12]. Likewise, the Hessian fly is also an introduced pest in New Zealand where it was first reported in the 1870s [9], [13], [14].

Hessian fly is a galling midge that is dependant on its host for survival. The primary host is wheat, although other closely related grass species are suitable when wheat is not available [15]. Due to its dependence on the host plant, this fly is able to manipulate cells within the host plant to induce a nutritive tissue, although it does not make a true gall [16]. The host plant may carry some form of resistance to Hessian fly, and thus, interacts on a gene-for-gene basis, similar to that of plant–pathogen interactions [17], [18], [19] (see Bent & Mackey [20] for a review of the gene-for-gene interaction). This interaction has been taken advantage of as a control measure by deploying numerous wheat varieties carrying various genes for resistance [9], [21], [22], [23], [24]. It has been generally thought that fly virulence to resistance genes in the plant comes only through homozygous recessive alleles, and therefore places selection pressure on flies in the field for recessive homozygotes [25]. As such, there has been a focus to identify and locate virulence genes in the Hessian fly [26], [27].

Despite the long time span since the introduction of Hessian fly into North America, we wanted to test if there is clear evidence of westward movement of Hessian fly colonization to support the rich historical accounts of a single introduction. Additionally, we wanted to test if we could identify a clear source population for either North America or New Zealand from currently sampled populations, as these locations represent two separate invasions.

## Methods

### Sample Collections, DNA Isolation, and Genotyping

It has been suggested that parts of West Asia may be the origin of Hessian fly, along with its host plant, and Hessian fly colonized Europe and North Africa 100 years prior to colonizing North America [6], [9], [28]. Therefore, all collections from Europe, North Africa, and West Asia (Old World), will be considered to be within the “native” range. While, unfortunately, we do not have samples from Germany or New York, as neither area recognizes a significant problem with Hessian fly, we still wanted to evaluate the relationships of Hessian fly populations between native and introduced areas that do recognize problems or where Hessian fly is common enough to collect.

Thirty collections of Hessian fly were obtained from nineteen states within the United States, one Canadian Province, and six non-North American countries (Table 1). Samples were sent in ethanol from landowners, and did not require permits. Ethanol-preserved adult flies or pupae were stored at −20°C, until DNA could be isolated and eluted in 100-µL elution buffer (DNeasy Tissue Kit, Qiagen Inc, Valencia, CA). Sex was not determined for pupal samples, and thus disregarded in any analyses. Eighteen polymorphic microsatellite loci of varying di-, tri-, and one tetra-nucleotide motifs included loci from all chromosomes, based on physical location from hybridization to polytene chromosomes [29] (Table 2). Due to the hemizygous nature of sex chromosomes in males [30] and the possibility of artificially increasing observed homozygosity, only three sex-linked loci were used. Polymerase chain reactions (PCR) were performed on each locus separately in 25 µl reactions containing 2.5 µl of 10X PCR buffer (Promega, Madison, WI), 1.5 µl of 25 nM MgCl_2_ (Promega), 5 µM dNTPs (Promega), 0.75 U of *Taq* DNA polymerase (Promega), 1.0 µl of reverse non-fluorescent primer (5 µM) and 0.5 µl of a fluorescently labeled forward primer (10 µM), labeled with one of three Beckman-Coulter dyes (Invitrogen, Carlsbad, CA), and 6–25 ng template DNA. Cycling conditions were 95°C for 4 min, 6 cycles of 95°C for 1 min, 50°C for 1 min, 72°C for 1 min, 31 cycles of 95°C for 30 sec, 50°C for 30 sec, 72°C for 55 sec, and a final extension of 72°C for 30 min performed in a MJ Research DNA Engine Dyad thermal cycler (MJ Research, Watertown, MA). After amplification, products were pool-plexed, which is the combining of loci for genotyping. The pool-plexes included 2 groups of nine loci, adding 2 µl of blue, 3 µl of green, and 12 µl of yellow/black dyed products. Genotyping mixes were made from 1 µl aliquot of pool-plexes, 0.5 µl of a 600 bp size standard (Beckman-Coulter), and 40 µl SLS buffer (Beckman-Coulter). Genotyping was then performed using a Beckman-Coulter CEQ8000, per manufacturer’s instructions, and sized with CEQ8000 software. Genotypes were checked for errors using Micro-Checker [31].

**Table 1:** Summary statistics for each collection and population.

Population		ID Code	n	H_O_	H_E_	D	N_A_	N_ea_	N_P_	*M*	F_IS_
North America											
NorthWest			96	0.428	0.542	0.55	5.17	2.75	0.167 (0.167)	0.997	0.22**
Oregon		OR	48	0.417	0.516	0.52	4.22	2.55		0.953	0.20**
Latah County, Idaho		LaID	48	0.438	0.539	0.55	4.39	2.65		0.970	0.20**
NorthCentral			156	0.451	0.568	0.57	6.22	2.69	0.278 (0.000)	0.945	0.21**
Teton County, Montana		TeMT	48	0.446	0.438	0.44	3.39	2.04		1.124	−0.01
Cass County, North Dakota		CaND	60	0.458	0.555	0.56	5.17	2.77		0.939	0.18**
Winnipeg, Manitoba, Canada		WiCa	48	0.447	0.565	0.57	4.83	2.68		0.898	0.22**
Central			298	0.447	0.585	0.59	8.56	3.04	0.944 (0.333)	0.939	0.24**
Randolph County, Illinois		RaIL	51	0.454	0.586	0.59	5.72	3.18		0.925	0.24**
Mississippi County, Missouri		MiMO	22	0.498	0.590	0.60	4.44	3.07		0.919	0.18**
Scott County, Kansas		ScKS	48	0.423	0.515	0.52	4.83	2.65		0.856	0.19**
Kay County, Oklahoma		KaOK	48	0.440	0.549	0.55	5.06	2.88		0.885	0.21**
McCulloch County, Texas		BrTX	48	0.434	0.519	0.52	4.39	2.47		0.811	0.17**
McLennan County, Texas		McTX	48	0.460	0.576	0.58	5.22	2.80		0.916	0.21**
Arkansas County, Arkansas		ArAR	33	0.449	0.600	0.61	5.72	3.30		0.918	0.27**
SouthEast			336	0.561	0.672	0.67	9.94	3.89	1.333 (0.556)	0.959	0.17**
Franklin Parish, Louisiana		FrLA	48	0.529	0.626	0.63	6.00	3.31		0.821	0.17**
Perry County, Alabama		PeAL	48	0.553	0.631	0.64	6.00	3.46		0.864	0.14**
Henry County, Alabama		HeAL	26	0.587	0.648	0.66	5.83	3.53		0.916	0.11**
Gadsden County, Florida		GaFL	48	0.575	0.634	0.64	6.22	3.54		0.998	0.10**
Sumter County, Georgia		SuGA	22	0.514	0.601	0.62	4.83	3.12		0.971	0.17**
Spalding County, Georgia		SpGA	48	0.580	0.599	0.61	6.06	3.04		0.980	0.04*
Lenoir County, North Carolina		LeNC	48	0.561	0.682	0.69	7.44	3.92		0.898	0.19**
Beaufort County, North Carolina		BeNC	48	0.577	0.661	0.67	6.56	3.69		0.880	0.14**
HoMS											
Holmes County, Mississippi		HoMS	48	0.414	0.426	0.43	4.39	2.20	0.000 (0.000)	0.882	0.04
FlSC											
Florence County, South Carolina		FlSC	48	0.586	0.599	0.61	5.06	2.94	0.167 (0.167)	0.945	0.03
EastCoast			66	0.549	0.634	0.64	6.33	3.16	0.056 (0.000)	0.870	0.143**
Richmond County, Virginia		RiVA	48	0.573	0.591	0.60	4.89	2.84		0.907	0.04*
Wicomico County, Maryland		WiMD	18	0.483	0.625	0.64	5.33	3.18		0.871	0.25**
Old World											
Morocco		Mo	32	0.434	0.572	0.58	5.22	2.67	0.500 (0.278)	1.018	0.26**
Spain		Sp	46	0.490	0.634	0.64	7.11	7.11	1.389 (0.667)	0.896	0.24**
Israel		IsA	21	0.361	0.475	0.49	3.94	3.94	0.667 (0.444)	1.377	0.26**
		IsB	15	0.470	0.500	0.52	4.28	4.28	0.444 (0.167)	0.794	0.1*
Syria		Sy	47	0.467	0.547	0.55	5.33	5.33	0.778 (0.389)	0.945	0.16**
Kazakhstan		Kz	47	0.390	0.517	0.52	5.44	5.44	0.667 (0.278)	1.012	0.26**
New Zealand		Nz	48	0.471	0.539	0.55	4.33	2.68	NA (0.111)	0.922	0.14**

Each population is listed along with the collection(s) that make up the population. North American populations are based on Tess analysis at K = 7 (see Figure 2 and text), and includes the identifier code for each population/collection (ID Code), number of individuals (n), observed heterozygosity (H_O_), expected heterozygosity (H_E_), Nei’s unbiased gene diversity (D), mean number of alleles per locus (N_A_), number of effective alleles (N_ea_), number of private alleles (N_P_), the value *M*, from a M-ratio test, and inbreeding coefficient (F_IS_), *denotes p-values<0.05 and **<0.0001.

**Table 2:** Motif, cytological location, and null allele frequency for each microsatellite locus.

Locus	Motif	Cytological Location	Mean null allele frequency
Hf24	(AGA)_8_	X2L	0.174
Hf70	(GTT)_9_	X1L	0.091
Hf73	(ACA)_7_	A1L	0.051
Hf97	(ACA)_6_	X1S	0.131
Hf101	(TTG)_5_	A2S	0.015
Hf102	(AAC)_9_	A1L	0.065
Hf103	(TC)_7_	A1L	0.036
Hf104	(AC)_8_	A2L	0.119
Hf108	(TTC)_15_	A1L	0.021
Hf109	(AGA)_5_	A1L	0.117
Hf112	(CAAA)_4_	A1L	0.081
Hf113	(CA)_19_	A1L	0.032
Hf114	(AAC)_7_	A1S	0.055
Hf116	(AG)_7_	A1S	0.042
Hf119	(TG)_9_	A1S	0.035
Hf124	(CA)_13_	A2S	0.141
Hf164	(AC)_7_	A2S	0.020
Hf174	(AC)_8_	A1L	0.024

### Genetic Diversity

For all applicable software, Create [32] was used to facilitate quick file preparation. Null allele frequencies were calculated for each locus using the software FreeNA [33]. Genotypic linkage disequilibrium was calculated between pairs of loci using the software LinkDos [34] based on the methods of Black & Krafsur [35]. Population descriptive statistics were calculated using GenAlEx v6.2 [36], with the exception of genetic diversity based on Nei’s unbiased genetic diversity (D) and the inbreeding coefficient (F_IS_), calculated in Fstat v2.9.3 [37], [38].

Three methods were used to search for a bottleneck to test if any signal of a bottleneck remained, either as a result of initial introduction or range expansion westward. First, a search for a recent population bottleneck was performed in Bottleneck [39], which uses the heterozygosity excess method [40]. For the bottleneck search, using the population designation found from population genetic structure analysis (detailed below), populations were run as follows: all populations, North America, and Old World. We employed a two-phase model with a 95% proportion of single-step mutations and a variance of 12, following Piry *et al*. [39] recommendations, and using 1,000 iterations. A Wilcoxon sign-rank test was used to evaluate the Bottleneck simulations for heterozygosity excess. A mode-shift test was also run to look for any distortion in the distribution of allele frequencies different than the typical L-shaped distribution, indicating a bottleneck [41]. Lastly, we calculated the M-ratio, which compares the number of alleles to their range in allele size (*M*) [42]. This method suggests that bottlenecks will decrease the number of alleles more quickly than the range in allele size, the recovery of which is correlated with post-population size and allows for identification of bottlenecks long after the bottleneck occurred. As recommended by the authors, using seven or more loci, a population at equilibrium will result in *M*>0.68, and should approach 1.

### Population Structure

To assess population genetic structure, we implemented a Bayesian clustering algorithm, which uses an explicitly spatial prior distribution, as utilized in Tess v2.3.1 [43], [44] to identify the number of clusters (K), which represents the number of populations. Tess has been shown to perform equally well, if not better, in discerning the number of populations as compared to other commonly used clustering software, especially if geographic admixture is moderate to low or if differentiation was weak [43], [45]. Despite some level of null alleles, all loci were used in assignment analyses because the influence of null alleles should not change the overall outcome of the assignment test, as explicitly tested by Carlsson [46]. Using geographic coordinates with microsatellite genotypes within Tess, 100 independent runs for each K were performed with 50,000 sweeps and a burn-in of 5,000 sweeps, and allowing for admixture. Samples were run in three groups (with the range of K values noted in parentheses): entire dataset (1 to 16), only North American samples (1 to 16), and only Old World samples (1 to 11). The Deviance Information Criterion (DIC) was used to compare model fits (those with a lower DIC fit better) by taking an average across the 100 independent iterations and plotting against K. Much like the plateau of log-likelihood values in Structure [47], when the DIC first reaches a plateau, that K-value indicates the number of clusters. Admixture estimates for the 10% of runs with the lowest DIC for each K-value were averaged in Clumpp version 1.1.2 [48] using the greedy option with 1,000 permutations. Distruct was used to visualize the results [49].

### Chromosomal-based Diversity

Diversity based on Nei’s unbiased genetic diversity was calculated for each locus in Fstat v2.9.3 [36], [37], and grouped according to chromosomal location; however, since there were only three sex-linked loci – two from X1, and one from X2 – all three loci were grouped together. Plots of diversity from all loci and from each chromosomal group were made between North America and the Old World to identify if a particular locus or chromosome exhibited a decreased level of diversity in North America.

Selection was also assessed for each locus by identifying loci with excessive or reduced F_ST_. In order to identify if there are any loci experiencing different selection pressures between North America and the Old World, these simulations were carried out with 1) the whole data set, 2) North American populations, and 3) the Old World populations, excluding New Zealand from simulations other than with the whole data set. Simulations were performed using the Fdist method [50], as implemented in Lositan [51]. Lositan was run for 50,000 simulations with both the infinite alleles model (IAM) and the stepwise mutation model (SMM), using both options for neutral and forced mean F_ST_, for each of the three groups tested.

## Results

### Population Diversity

With a few exceptions, null allele frequencies across loci were generally low (Table 2). Loci located on the sex chromosomes tended to have a higher frequency of null alleles compared to autosomal loci. This could be attributed to the hemizygous state of sex chromosomes in males, and thus, an over estimate of homozygosity at those loci [30]. No significant linkage was detected, so all loci were considered independent markers. Diversity across all populations was comparable for all summary statistics within North America (Table 1). Comparing North American and Old World groups, there were no significant differences between most diversity statistics. Based on the Wilcoxon sign-rank test for heterozygote excess and the mode-shift test, employing a two-phase model, no populations were found to show evidence of a bottleneck. The M-ratio test returned *M* values all greater than 0.68, and all values around 1, indicating equilibrium (Table 1).

### Population Structure

**Figure 1:**
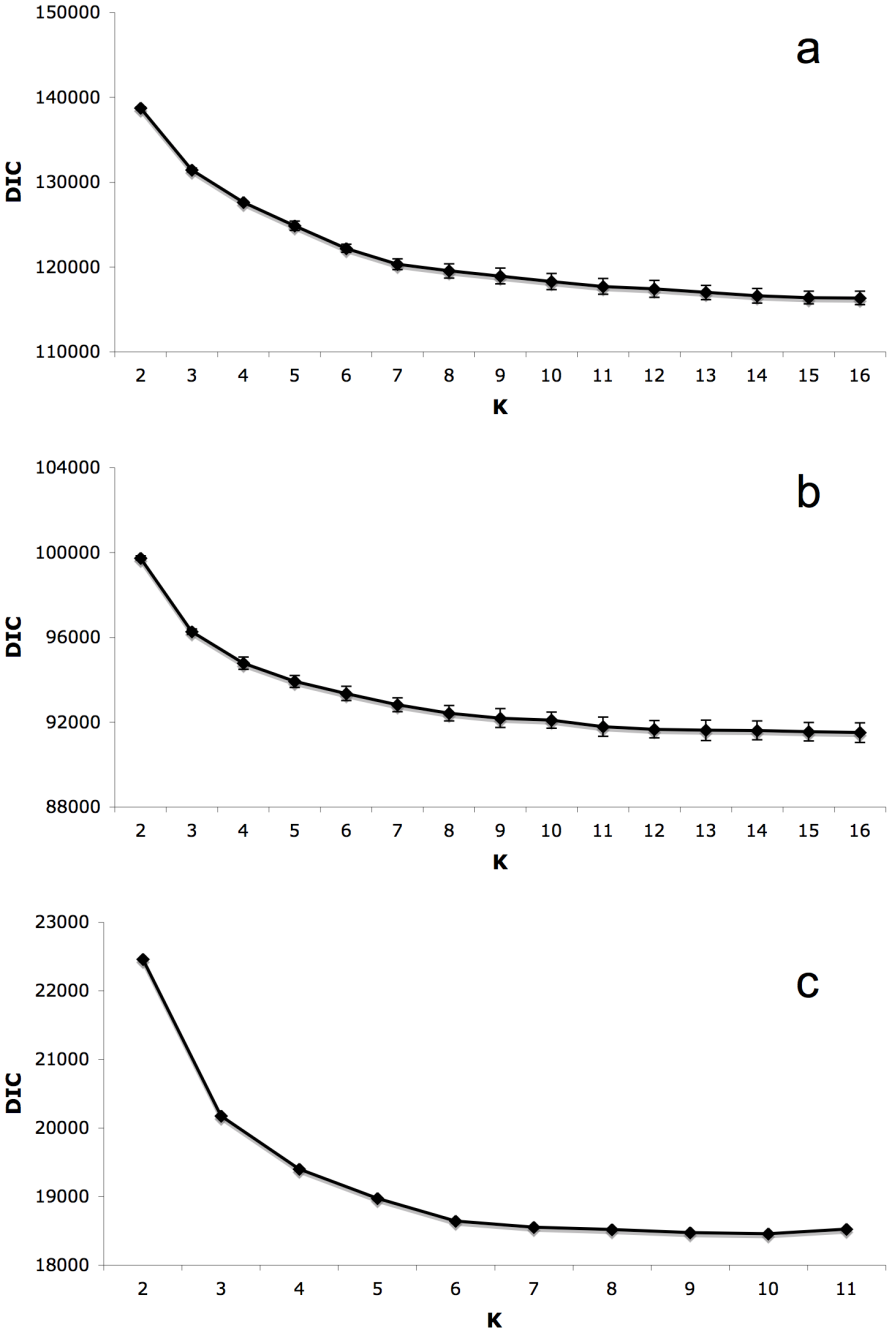
Deviance Information Criterion (DIC) plots. Plot of DIC values against K to estimate an inverse “plateau” that is representative of the actual K for each of the three independent Tess runs: (a) the entire data set, (b) North American collections only, and (c) Old World collections only.

**Figure 2:**
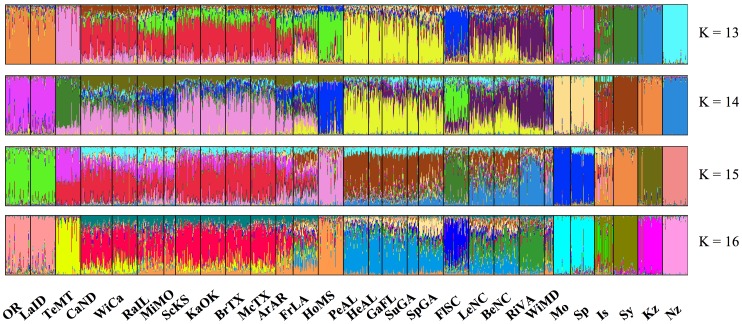
Bar plots of admixture assignments for the entire data set, spanning North America, Old World, and New Zealand collections, based on Bayesian clustering implemented in Tess, showing K = 13–16. Each bar represents a single individual with the colors indicating the likelihood assignment of the individual to an inferred genetic cluster. Location abbreviations are the same as in Table 1.

**Figure 3:**
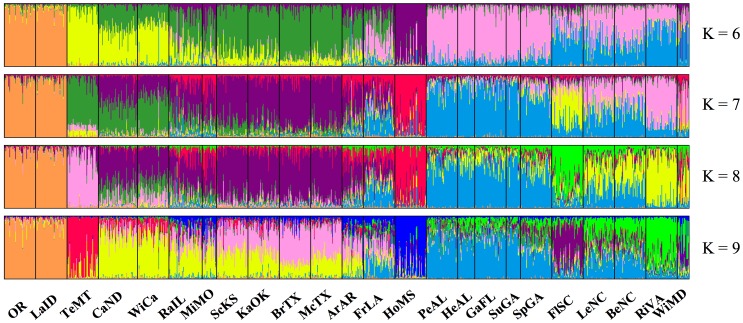
Bar plots of admixture assignments for North American collections, based on Bayesian clustering implemented in Tess, showing K = 6–9. Bars and abbreviations are representative as stated in Figure 1.

**Figure 4:**
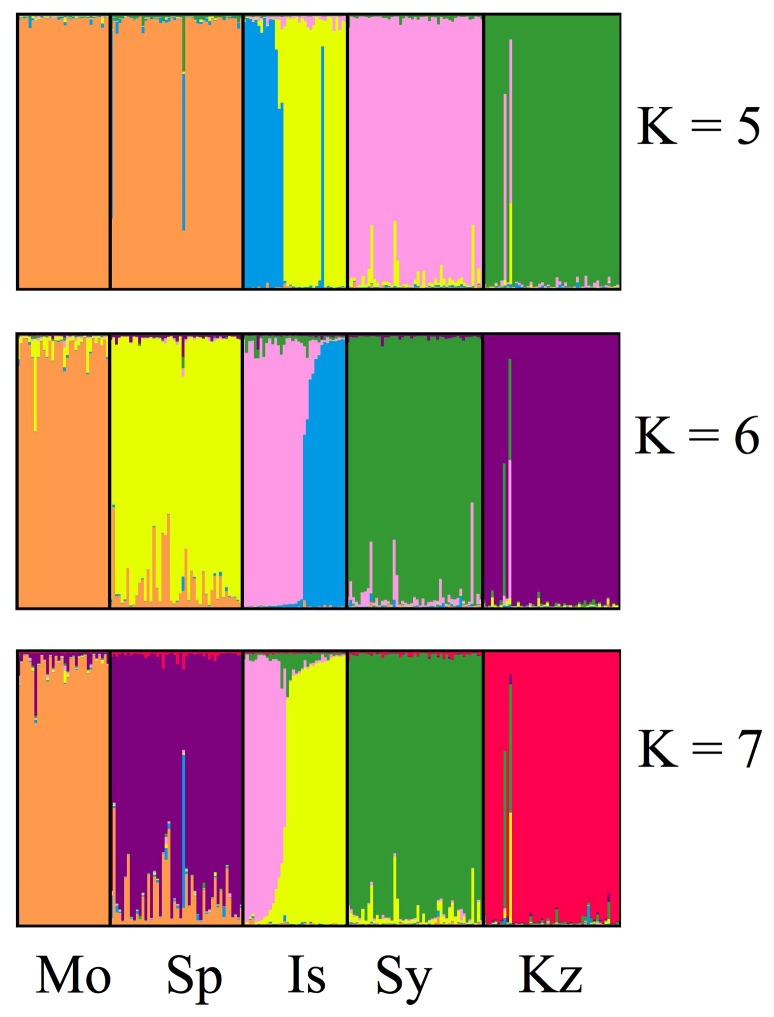
Bar plots of admixture assignments for Old World collections, based on Bayesian clustering implemented in Tess, showing K = 5–7. Bars and abbreviations are representative as stated in Figure 1.

In Tess, using the entire data set, DIC values indicated a plateau at 14 clusters as representative of the number of populations for all samples (Fig. 1a and 2). Each of the Old World populations fell into distinct clusters, with the exception of Spain and Morocco clustering together. New Zealand did not cluster with any other population. When run independently of other samples, DIC values indicated a plateau at 7 clusters (Fig. 1b and 3). Clustering was similar for North American collections when run both independently and with the entire data set. Regional clusters were dominant; however, two collections maintained their own unique population identity, namely HoMS and FlSC (Holmes County, MS and Florence County, SC – see Table 1). The collections that fell into regional clusters are depicted in Table 1. To further inspect population genetic structure, K = 8 was also investigated. Upon inspecting individual cluster assignment, again, only 7 clusters were ever found which an individual had a majority assignment. Furthermore, regional clustering was not influenced by an increase in K, with the exception that TeMT clustered uniquely, and CaND and WiCa joined the Central cluster (Teton, MT; Cass, ND; Winnipeg, Canada; and the Central population, respectively – see Table 1). For all further North American analyses, K = 7 was used. When Old World collections were run independently, a DIC plateau at K = 6 was found (Figure 1c and 4). Each collection formed its own distinct population, with Spain and Morocco separated. Additionally, two distinct populations were found within the Israeli collection, despite these flies being collected from the same location.

### Chromosomal-based Diversity

**Figure 5:**
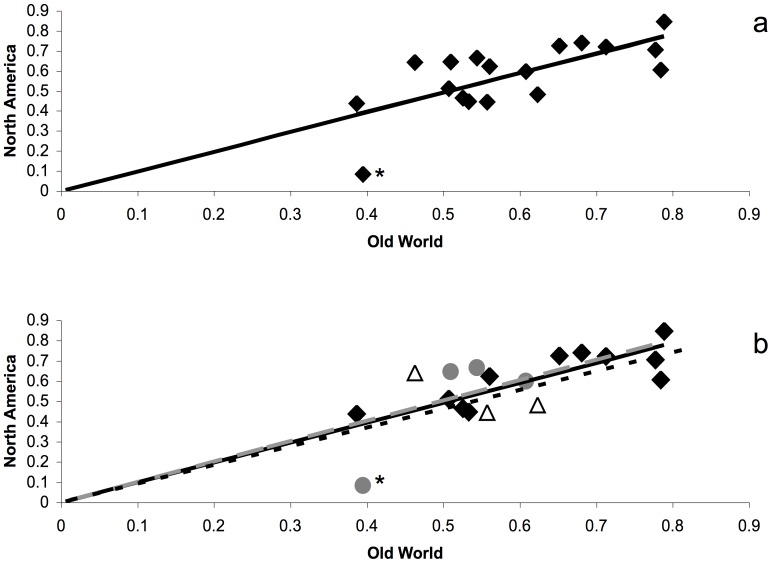
Locus and chromosomal-based diversity plots. Diversity plots from individual loci between North America and the Old World, excluding New Zealand, with the (a) entire data (R^2^ = 0.484), and (b) with the loci divided into the chromosomal groups of A1 (black diamonds and solid black trendline, R^2^ = 0.660), A2 (gray circles and dashed gray trendline, R^2^ = 0.438), and combined X1 and X2 (white triangles and dotted black trendline, R^2^ = −1.733). Asterisk denotes locus Hf101.

Little variation was found in the assessment of diversity between North America and the Old World (Fig. 5). Comparing all loci between North America and the Old World, a linear regression analysis found a line with a slope of 0.9817 (R^2^ = 0.4839), very close to a neutral slope of 1.0, indicating among all loci, diversity in North America does not drastically differ from that of the Old World (Fig. 5a). Additionally, when loci are grouped according to chromosome, their slopes are as follows: A1 = 0.9857 (R^2^ = 0.6604), A2 = 1.0088 (R^2^ = 0.4384), X1 and X2 combined = 0.9292 (R^2^ = −1.733) (Fig. 5b). Therefore, in a comparison between North America and the Old World, individual chromosomes experience neither an increase, nor a decrease in diversity. One locus, Hf101, was observed as an outlier, located on chromosome A2, which had a reduction in diversity from 0.40 in the Old World to 0.10 in North America. Many North American populations were monomorphic at locus Hf101, leading to this reduction.

The only loci identified as candidates for selection were Hf119 and Hf174. Both loci were identified as being under balancing selection under the IAM only. Under the SMM, no loci were identified as being under any selection. This result was the same for the North American populations (both in K7 and K8), as well as the Old World populations. Both loci under selection were located on chromosome A1; however, Hf119 is located on the short arm and Hf174 on the long arm [29].

## Discussion

All Old World collections consisted of single, independent populations, with the exception of the Israeli collection containing two distinct populations. Finding two populations within the Israeli collection is very interesting as these flies were all collected within the same field, yet have a very distinct population genetic structure. This result may not be entirely surprising as Israel is closely located to the putative origin of wheat and Hessian fly, and many species of wild wheat natively occur within the country [6], [9], [28]. Despite their close geographic proximity, the vast difference in these two Israeli populations is surprising, especially compared with populations in North America. For example, collections in North America could be several states away, yet still are included in the same population while these two Israeli populations are sympatric but maintain distinct populations. Previous work has shown similarly striking results with respect to the differentiation of Israeli Hessian fly mitochondrial haplotypes to the rest of the world [52], as well as differentiation within Israel [53]. This result certainly lends itself to asking more specific questions about the population genetic relationships of Hessian flies from Israel, as well as the rest of the area, such as the possibility of incipient speciation.

None of the introduced populations from North America or New Zealand clustered together with any of the Old World populations (Fig. 2). This result was not entirely unexpected since a representative collection from Germany was not available, and 200 years have passed since the putative introduction of Hessian fly into North America. Additionally, we can assess that the New Zealand population did not directly originate from any of these Old World or North American populations. Clustering results also show that the western populations are distinctly different from Spain. Even if there was a Spanish introduction of Hessian fly to the west coast, they have diverged significantly. Incorporating samples from California would be the best test of this hypothesis.

In North America, seven populations were found across a majority of the wheat-growing region, with more Hessian fly collections sampled than in any prior study. All collections fit into pseudo-regional populations, with the exception of FlSC and HoMS, which stood independently as their own population. With respect to the populations in the southeastern US, overall similar results were found previously [54]; however, the additional collections added to the resolution of the population genetic structure. For example, Morton et al. [54] found K = 2 in the southeastern populations, although there was more structure when looking at a higher K value. In the current study, however, a higher K value was found, further refining the population genetic structure in the southeastern US and providing similar results as the higher K values (e.g. K = 4) in Morton et al. [54]. These results suggest divergence between different regions with a higher level of gene flow within a region. The population genetic structure of North America very closely matches the regional association of wheat class data (http://webarchives.cdlib.org/sw15d8pg7m/http:/ers.usda.gov/Briefing/Wheat/maps.htm), as suggested by Black et al. [12] and Morton et al. [54]. This suggests that populations could be closely associated with a local adaptation to a particular wheat class, although this hypothesis remains to be explicitly tested.

We found similar genetic diversity between all Hessian fly populations, regardless of their origin; however, when observing those populations that are centrally located in North America, such as Central and SouthEast, we observed a slight decrease in diversity and allelic richness in the populations as the distance from the coast decreased. This could be due to the fact that these centrally located populations were made up of more collections; however, it could also be an indication that genetic mixing commonly occurs in these areas. Unfortunately, this pattern of slightly higher diversity in the centrally located populations, and decreasing moving out, neither expressly lends itself to providing evidence of a westward expansion, nor does it refute the possibility. Private alleles were found in the most westward populations (Table 1), either adding to the evidence against westward expansion or indicating more sampling is necessary. Additionally, no evidence for a genetic bottleneck was present, as might be expected with a signal of westward expansion. Previous work has shown populations in the northwest have a reduced mitochondrial haplotype diversity [52], which this reduction in diversity does not seem to be shared to the same extent with the nuclear genome. Packard’s theory of a separate introduction of Hessian fly from Spain to California, resulting in Hessian fly on the west coast [10], is a scenario that could provide the private alleles we see in our NorthWest and NorthCentral populations, as well as support the pattern we observe with a “peak” of diversity/private alleles in the Central and SouthEast populations (see Table 1 for population descriptions).

As a test to identify if there had been any increasing and/or differing selection pressures in North America compared with our Old World samples, we compared genetic diversity at each locus and for each chromosome (Fig. 5). This comparison could provide additional information on key loci that may help in revealing a signature of westward expansion. One expectation was that while diversity was constant between populations, one or two loci between North America and the Old World might be experiencing different pressures. This expectation was due to more resistant wheat varieties readily available in the United States compared to other areas around the world [23]. In comparison between the diversity at each locus and each chromosome, there were few differences (Fig. 5). The only locus that did seem to have a difference was Hf101. This locus, however, was mostly monomorphic in the North American populations, which could be the result of stochastic fixation in addition to any potential selection effects. Furthermore, this locus was not found to have any selection pressures based on the results from Lositan. The lack of differences in diversity on a chromosomal basis between the North American and Old World groups is supported by the findings that both the North American and Old World populations have the same two loci under selection. This indicates, that despite over 200 years of separation and the high probability of differing selection pressures in the field [25], the loci tested here do not show differences in selection. It is not surprising to find that the loci under selection exhibit balancing selection. Due to the gene-for-gene interaction between the Hessian fly and wheat [9], [21], [22], [23], [24] and the great potential for fluctuating selection (from changes in wheat varieties planted over time and space), it is reasonable to think that balancing selection is common. This would be due to the potential for obviation, or the survival of heterozygous larvae on the same host plant as homozygous larvae [55], as potentially only the homozygous recessive alleles in the fly confer virulence on a resistant wheat plant [25]. Selection pressure from resistant wheat varieties, coupled with obviation, and the unusual chromosome inheritance of Hessian fly [30], could also account for nearly all populations exhibiting significant F_IS_ values, and therefore contribute to these populations not maintaining Hardy-Weinberg equilibrium. Inbreeding, however, may exist in these populations, and the population structure could be an artifact of common descent, but a higher resolution of loci would be required to test this hypothesis.

The minimal evidence of a westward movement, along with the lack of differences in diversity and private alleles between North America and the Old World is consistent with evidence of multiple introductions [56]. For most invasive species, a lower genetic diversity in the introduced area is considered typical [56], [57]. For Hessian fly, this type of scenario would have shown a lower genetic diversity in the eastern and southern populations with decreasing diversity as populations move west, due to the multiple founding populations during westward expansion. Evidence provided here suggests there has been significant divergence between North American and Old World populations, such that introduced populations no longer resemble any of the Old World populations. Furthermore, this is supported by relatively little evidence of a westward expansion, as has been historically purported as the invasion scenario of Hessian fly in North America as a single incidence. The unfortunate gaps in our sampling include the lack of collections of Hessian fly from New York and Germany, which does inhibit an exhaustive evaluation of these hypotheses. These samples remain missing pieces to this puzzle, as Hessian fly does not seem to pose a significant or substantial threat to these areas any more, making sample collection difficult.

In this study we did not find overwhelming support of a westward expansion, although this result adds to uncovering the invasion history of one of North America’s oldest invasive insects. We did observe that after >200 years, a significant proportion of population admixture still remains in North America, compared to Old World counterparts. It will be interesting, however, to further explore if the population structure in North America is a result of an association between populations and wheat class, or some other factor, adding to the development of hypotheses on the biology of this invasive insect. Furthermore, our data adds to the possibility of a multiple invasion scenario, and due to the methods employed here, provides a baseline for the quantity of data necessary to reconstruct the history further. As the signal of invasion has weakened over the last >200 years, future investigations of Hessian fly may need to be conducted to examine this invasion scenario on a genomic level to further tease apart this dramatic history.
